# White and Grey Matter Changes in the Language Network during Healthy Aging

**DOI:** 10.1371/journal.pone.0108077

**Published:** 2014-09-24

**Authors:** Yanhui Yang, Bohan Dai, Peter Howell, Xianling Wang, Kuncheng Li, Chunming Lu

**Affiliations:** 1 Department of Radiology, Xuanwu Hospital, Capital Medical University, Beijing, P.R. China; 2 Key Laboratory for Neurodegenerative Diseases, Ministry of Education, Beijing, P.R. China; 3 State Key Laboratory of Cognitive Neuroscience and Learning & IDG/McGovern Institute for Brain Research, Beijing Normal University, Beijing, P.R. China; 4 Center for Collaboration and Innovation in Brain and Learning Sciences, Beijing Normal University, Beijing, P.R. China; 5 Division of Psychology and Language Sciences, University College London, London, United Kingdom; 6 Department of Neurology, Xuanwu Hospital, Capital Medical University, Beijing, P.R. China; 7 Beijing Key laboratory of Magnetic Resonance Imaging and Brain Informatics, Beijing, P.R. China; University of Leipzig, Germany

## Abstract

Neural structures change with age but there is no consensus on the exact processes involved. This study tested the hypothesis that white and grey matter in the language network changes during aging according to a “last in, first out” process. The fractional anisotropy (FA) of white matter and cortical thickness of grey matter were measured in 36 participants whose ages ranged from 55 to 79 years. Within the language network, the dorsal pathway connecting the mid-to-posterior superior temporal cortex (STC) and the inferior frontal cortex (IFC) was affected more by aging in both FA and thickness than the other dorsal pathway connecting the STC with the premotor cortex and the ventral pathway connecting the mid-to-anterior STC with the ventral IFC. These results were independently validated in a second group of 20 participants whose ages ranged from 50 to 73 years. The pathway that is most affected during aging matures later than the other two pathways (which are present at birth). The results are interpreted as showing that the neural structures which mature later are affected more than those that mature earlier, supporting the “last in, first out” theory.

## Introduction

Dealing with the effects of healthy aging is one of the biggest challenges facing the world today. Studies have shown that aging brings with it significant changes in white and grey matter (WM and GM) architecture that serve various functions [1], [2]. The neural changes that happen during aging have been explained by the “last in, first out” (LIFO) theoretical principle [3], [4]. Studies have shown that the late-myelinating neocortical regions are most vulnerable to aging and aging-related disease, whereas the primary motor and sensory regions that myelinate early are more resistant to these changes, only being affected at later ages [5], [6]. Consistent with this, a recent large-scale longitudinal volume examination of both neural development and aging confirmed that cortices that develop late are especially vulnerable to atrophy in aging [7]. However, there is also evidence showing that aging-related WM change occurs in specific fiber bundles that do not follow LIFO [8]–[10]. Thus, alternative principles such as “first in, first out” (FIFO) are also plausible. According to FIFO, the neural structures that mature earlier would be the first to be affected after maturity.

It is well known that myelination can markedly increase speed of signal transmission, which is important for the integration of information across the highly distributed neural networks that underlie higher cognitive functions such as memory, executive functions, and language. Aging is associated with reduction of length of myelinated axons, which might have a marked impact on those higher cognitive brain functions that are most susceptible to these changes [3]. Here aging-related neural structure changes in the language network were examined as this has received little attention.

It is well established that areas of the brain that deal with language are connected by: 1) A ventral pathway between the mid-to-anterior superior temporal cortex (STC) and the ventral inferior frontal cortex (IFC) and insula traversing the extreme capsule; and 2) Two dorsal pathways passing via the arcuate fasciculus and the superior longitudinal fasciculus (SLF). One of these two pathways connects the mid-to-posterior STC (Wernicke's area) with the premotor cortex, and the other one connects Wernicke's area with the IFC (Broca's area) via the premotor cortex [11]–[14]. Recent evidence has shown that the ventral pathway is present at birth as is the dorsal pathway that connects the STC and the premotor cortex [15]. However, the dorsal pathway that connects the STC and the IFC is not detectable in newborns [15] and appears to mature late [16].

A cross-sectional design was used to examine how the neural structures for language that mature at different ages are affected during healthy aging. Participants' ages covered a 24-year range (from 50 to 70+ years). Two independent groups of participants were recruited. The data from the second group were used to validate the results obtained from the first group. Both fractional anisotropy (FA) of WM fibers and cortical thickness of GM were examined to establish the relationship between WM and GM changes during aging. LIFO would predict that neural structures that lie in the dorsal pathway connecting the STC with the IFC would be affected more than those that lie in the other two pathways during aging, whereas FIFO would predict the opposite pattern.

## Methods

### Participants

Two groups of right-handed native Mandarin speakers were recruited. The first group included thirty-six participants whose ages ranged from 55 to 79 years (Mean  =  63 years, S.D. = 6.34, 16 females). The second group included twenty participants whose ages ranged from 50 to 73 years (Mean  =  61 years, S.D. = 7.23, 9 females). The large age range made correlation analysis between age and neural structure indexes feasible. All participants had hearing loss within age-appropriate ranges, and eyesight deterioration was corrected by glasses. The amount of education participants received ranged from 3 to 17 years (Mean  =  10 years, S.D. = 3.28) for the first group and from 8 to 14 years (Mean  =  12 years, S.D. = 1.97) for the second group. In order to exclude potential cognitive impairment, all participants were assessed by the Mini Mental State Examination (MMSE) [17]. The MMSE scores indicated that the cognitive functions of these participants were all within the normal range (group one: from 24 to 30, Mean  =  28, S.D. = 1.61; group two: from 28 to 30, Mean  =  29, S.D. = 0.74). Additional clinical assessment also showed that no participant had cognitive or neurological problems. All participants were right-handed, as assessed by the Edinburgh Handedness Inventory [18].

### Ethics Statement

Written informed consent was obtained from each participant. The study protocol was in compliance with the Code of Ethics of the World Medical Association (Declaration of Helsinki) and was approved by the Research Ethics Committee of Xuanwu Hospital, Capital Medical University.

### Imaging data acquisition

Imaging data were acquired from all participants on a Siemens TRIO 3T scanner at Xuanwu Hospital. Participants lay supine within the scanner with their head secured with foam padding. Structural images were obtained first from each participant with a high resolution T1-weighted MP-RAGE sequence. Then, the DTI images were obtained using an echo planar imaging (EPI) sequence. The scanning parameters are given in the Supporting Information (Text S1).

### Imaging data analysis

The two groups were not compared directly because the scanning parameters differed. Regions-of-interest (ROIs) identified from the whole-brain analyses of the first group were examined in the data from the second group.

### Analyses of group one

#### DTI data analysis

All DTI images were processed following the TBSS pipeline which is part of the FMRIB Software Library (FSL, http://www.fmrib.ox.ac.uk/fsl). Briefly, images were preprocessed to correct for motion and eddy current distortion, and the diffusion tensors were fitted to each voxel. The measure of FA was derived voxel-wise. The TBSS registration then nonlinearly transformed the FA images using the FMRIB58_FA standard space image (http://www.fmrib.ox.ac.uk/fsl/data/FMRIB58_FA.html) as the target, and the tract skeletonization process was subsequently performed using a threshold FA of 0.2 to exclude non-WM voxels. The final WM skeleton is a representation of the WM tract geometry common to the entire group of participants.

Whole-brain FA skeletons were regressed with age using a general linear model (GLM) method. Sex and education were included as covariates. Statistical parametric maps were thresholded at a voxel-wise level of *P*<0.05 (corrected by Monte Carlo simulation method, individual voxel *P*<0.01, cluster size> 13 mm^3^). The Monte Carlo simulation used the 3dCustSim program of AFNI (Analysis of Functional NeuroImages, see http://afni.nimh.nih.gov/pub/dist/doc/program_help/3dClustSim.html) on the WM skeleton image [19], [20].

#### Cortical thickness analysis

Cortical surface reconstruction and thickness measurements were performed using the FreeSurfer toolkit (http://surfer.nmr.mgh.harvard.edu/). Briefly, the structural image data of each participant were motion-corrected and the volume data were obtained. Cerebral GM/WM was then segmented and the GM/WM boundaries were estimated [21]. Topological defects at the GM/WM boundaries were corrected [22]. The boundaries were then used in a deformable surface algorithm designed to find the pial surface with submillimeter precision [23]. Cortical thickness measurements were obtained by calculating the distance between the GM/WM boundary and pial surface at each of approximately 160,000 points (per hemisphere) across the cortical mantle [23].

The surface representing the GM/WM border was “inflated” [24], differences among individuals in the depth of gyri and sulci were normalized, and each participant's reconstructed brain was then morphed and registered to an average spherical surface representation that optimally aligned sulcal and gyral features across participants [24], [25]. Thickness measures were then mapped to the inflated surface of each participant's reconstructed brain [24]. The data were smoothed on the surface using an iterative nearest-neighbor averaging procedure with a full width at half maximum of 10 mm. The data were then resampled into a common spherical coordinate system [25]. Further details of the method are available elsewhere [21], [23], [24], [26], [27].

A surface map was generated by regressing the cortical thickness with age using a GLM method (*P*<0.05, corrected by Monte Carlo simulation method on the segmented GM image, individual voxel *P*<0.01, cluster size> 100 mm^2^). Sex and education were included as covariates.

#### ROI validation of the aging-related neural changes

Because the correlation in the whole-brain analysis might be slightly above threshold in one region but slightly below threshold in another region, it was possible that this would lead to spurious difference in correlations among regions. In order to address this issue, a regression analysis was conducted on the WM/GM regions, where whole-brain analysis showed significant results, to test for differences in age correlations across regions. The analysis was conducted using IBM SPSS software (version 20, http://www.ibm.com/software/analytics/spss/).

The analysis involved the following steps. First, two groups of ROIs were selected: 1) Brain regions where significant correlations with age in WM/GM were found in the whole-brain analysis (see *Results*); 2) Two brain regions in the ventral pathway were selected as controls that mature earlier than the target dorsal pathway. The first control region was a WM control region in the extreme capsule underlying the anterior STC [EC-STC, x, y, z = −43, −5, −24 in Montreal Neurological Institute (MNI) template, radius =  3 mm]. This was located according to ICBM-DTI-81 atlas [28], [29] that is distributed with FSL software. The second control region was a GM control region in the anterior STC (x, y, z = −49, −5, −24 in MNI template, radius  =  3 mm) located according to the spherical coordinate system in Freesurfer [25]. Cortical thickness or FA values were then extracted from these ROIs for each participant, and transformed into Fisher's *z*-values. Then a regression analysis was conducted, in which all WM ROIs and the interactions between these WM ROIs were included as independent variables (see *Results*), and age was the dependent variable. A similar analysis was conducted on the GM ROIs. Finally, the results were corrected at *P*<0.05 level using a false discovery rate (FDR) method (FDR toolbox, http://www-personal.umich.edu/~nichols/FDR/, running under MatLab, http://www.mathworks.cn/products/matlab/). It was hypothesized that if LIFO applies, the ROIs that lie in the dorsal language pathway connecting the STC with the IFC should make a significant contribution to the change with age, but the ROIs that lie in the other two language pathways should not. FIFO predicts the opposite pattern.

#### Consistency among WM/GM changes

To establish whether different brain regions showed similar patterns of neural change with age, the WM ROIs were correlated with each other. The same procedure was conducted on the GM ROIs. The results were corrected at *P*<0.05 level using the FDR method. It was hypothesized that the control WM/GM ROIs would show different patterns of neural changes with age to those seen in the target ROIs. That is, the neural structural changes in the control ROIs would neither correlate significantly with those seen in the target ROIs nor with age.

#### Consistency between WM and GM changes

Correlations between each WM ROI and each GM ROI were computed to examine the consistency between WM and GM changes with age. The results were corrected at *P*<0.05 level according to the FDR method.

The following additional analyses were conducted in order to further elucidate the relationship between WM and GM changes and to specify which pathways were most affected during aging. First, FA values from all WM ROIs and cortical thickness from all GM ROIs (except for the control ROIs) were averaged separately to generate a grand-averaged FA value (GA-FA) and a grand-averaged cortical thickness value (GA-thickness). The correlation between GA-FA and GA-thickness was computed to examine the general relationship between GM and WM. Second, FA values from WM ROIs in the left (except for the control ROI of the EC-STC) and right hemispheres were averaged separately to generate a left FA value (L-FA) and a right FA value (R-FA). The L-FA and R-FA were correlated to the GA-thickness in order to distinguish the relationship between GM and WM changes in the left and right hemisphere respectively. Third, the ROIs that might be distinctive for, or have a close relationship with, the late-maturing fibers underlying the left IFC, were combined to generate the anterior part (L-A-FA) of the dorsal pathway. The ROIs in the temporal-parietal association cortex were considered as the posterior part (L-P-FA) of the dorsal pathway (see *Results*). L-A-FA and L-P-FA were correlated to GA-thickness to determine the roles of the two parts of the dorsal pathway. In order to confirm the correlation analysis, a regression analysis was conducted. In this analysis, the GA-thickness was predicted by the L-A, R-FA, L-A-FA, L-P-FA, and interactions between L-A-FA and L-P-FA.

In order to further confirm the relationship between L-FA, R-FA, L-A-FA, L-P-FA and age, an additional regression analysis was conducted that used age as the dependent variable.

### Re-examination of the neural changes in group two

In order to validate the results obtained from group one, the ROIs selected from this group were used as masks to extract both FA and cortical thickness for group two. The analysis procedures outlined above were conducted using group one's ROIs with group two's data.

## Results

### Whole-brain DTI results

**Figure 1:**
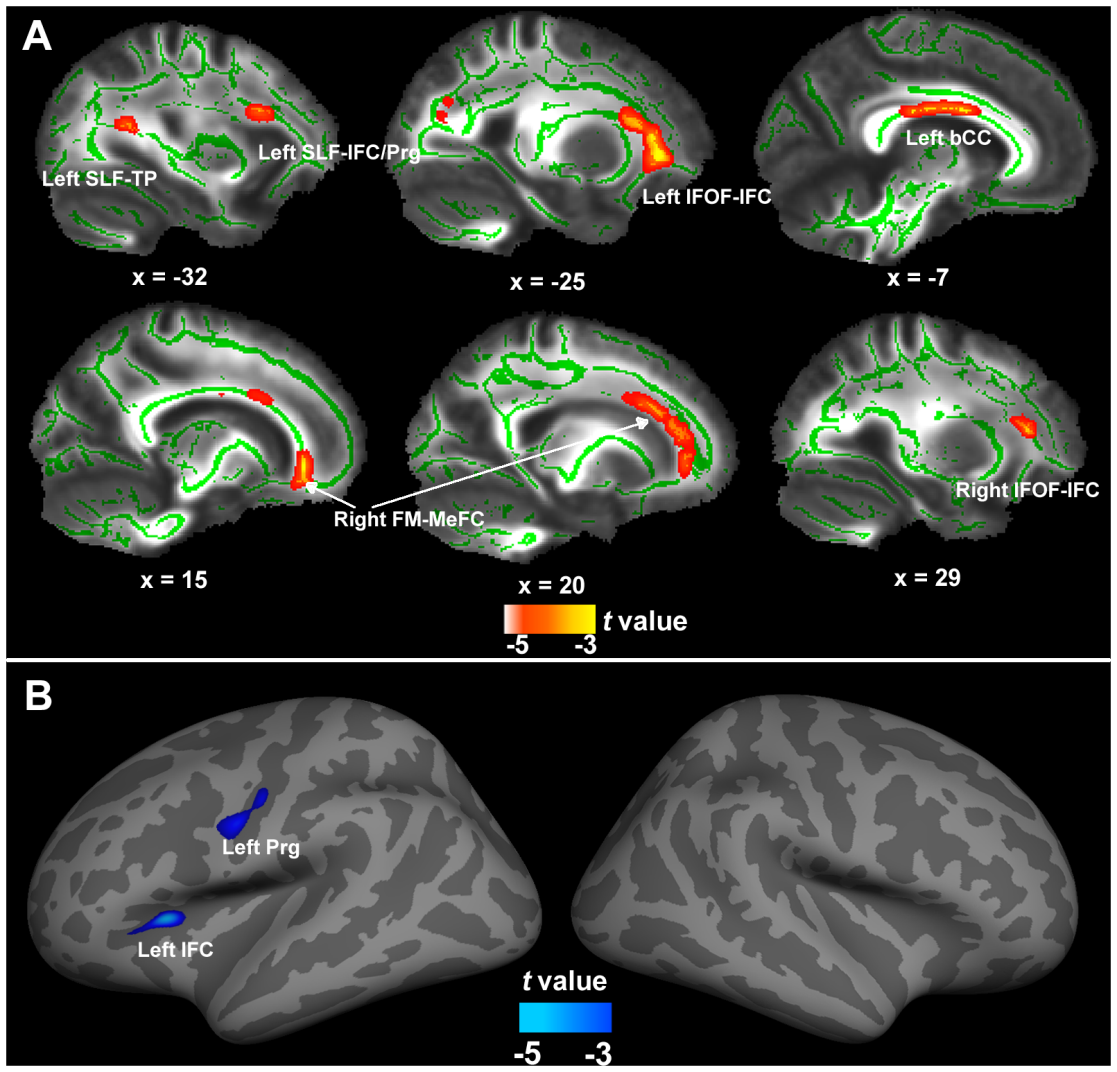
Neural structural changes with age in Group 1. (A) shows FA changes. Note that the skeletonized results are “thickened” to help visualization. Left SLF-IFC/Prg, left superior longitudinal fasciculus underlying the inferior frontal cortex and precentral gyrus; Left SLF-TP, left superior longitudinal fasciculus underlying the temporal-parietal association cortex; Left and right IFOF-IFC, left and right inferior fronto-occipital fasciculus underlying the inferior frontal cortex; Right FM-MeFC, right forceps minor underlying the medial frontal cortex; Left bCC, left body of corpus callosum. (B) shows cortical thickness changes. The colored blobs (blue for cortical thickness, red for FA) indicate brain areas that correlated negatively with age. Left IFC, left inferior frontal cortex; Left Prg, left precentral gyrus. No positive correlations were found.

In accord with the expectations, there were two brain regions along the late-maturing dorsal pathway that connects the STC with the IFC that showed significant negative correlation with age: One underlies the IFC and precentral gyrus (SLF-IFC/Prg), and the other one underlies the temporal-parietal association cortex (SLF-TP). In addition to these, significant negative correlations were also found in the bilateral inferior fronto-occipital fasciculus underlying the IFC (IFOF-IFC), right forceps minor underlying the medial frontal cortex (FM-MeFC), and the body of the corpus callosum (bCC). Table 1 and Figure 1A summarize these results.

**Table 1:** Brain regions showing significant correlations with age in group one.

Brain regions	Position			t-value	Volume (mm^3^/mm^2^)
	x	y	z		
**White matter (FA) changes**					
Left inferior fronto-occipital fasciculus underlying the inferior frontal cortex	−25	33	8	−7.36	634
Left superior longitudinal fasciculus underlying the inferior frontal cortex and precentral gyrus	−32	15	22	−5.88	142
Left superior longitudinal fasciculus underlying the temporal-parietal association cortex	−31	−47	16	−4.185	139
Left body of corpus callosum	−7	3	26	−5.164	465
Right inferior fronto-occipital fasciculus underlying the inferior frontal cortex	29	31	12	−6.704	213
Right Forceps minor underlying the medial frontal cortex	15	34	−4	−5.491	154
	20	21	29	−3.656	153
**Grey matter (cortical thickness) changes**					
Left inferior frontal cortex (pars triangularis, BA47)	−31	21	12	−3.199	165
Left precentral gyrus (BA4)	−43	−14	29	−2.614	331

No significant positive correlations were found in either the left or right hemisphere, nor in the extreme capsule of the ventral pathway.

### Whole-brain cortical thickness results

Two brain regions along the late-maturing dorsal pathway correlated negatively with age. One was located in the left IFC (pars triangularis, BA47) and the other in the precentral gyrus (Prg, BA4). No significant positive correlations were found (Table 1 and Figure 1B). These results confirmed the DTI results, indicating that brain regions along the dorsal pathway that connects the STC with the IFC showed significant decline of cortical thickness with age, particularly the part that extends to the IFC.

### ROI validation of the aging-related neural changes

The first regression analysis included all WM ROIs and their interactions (Left: SLF-IFC/Prg × SLF-TP, SLF-IFC/Prg × IFOF-IFC, SLF-TP × IFOF-IFC, SLF-IFC/Prg × SLF-TP× IFOF-IFC; Right: IFOF-IFC × FM-MeFC) as independent variables. The resultant regression model (*F*_(2,35)_ = 32.873, *P*<0.001) showed that the left SLF-IFC/Prg (*β* = −0.368, *P* = 0.026) and IFOF-IFC (*β* = −0.498, *P* = 0.003) accounted for almost 67% of the variance over age (*R^2^* = 0.666). No other variables made significant contributions. The second regression analysis included all GM ROIs and their interactions (IFC, Prg and IFC × Prg). The resultant regression model (*F*_(2,35)_ = 13.795, *P*<0.001) showed that the IFC (*β* = −0.447, *P* = 0.003) and Prg (*β* = −0.369, *P* = 0.011) accounted for 46% of the variance with age (*R^2^* = 0.455). No other variables made significant contributions. These results indicated that neural changes in both GM and WM of the dorsal pathway connecting the STC with the IFC made significant contributions to the variance with age compared to those in other anatomical positions.

### Consistency among WM/GM changes

**Figure 2:**
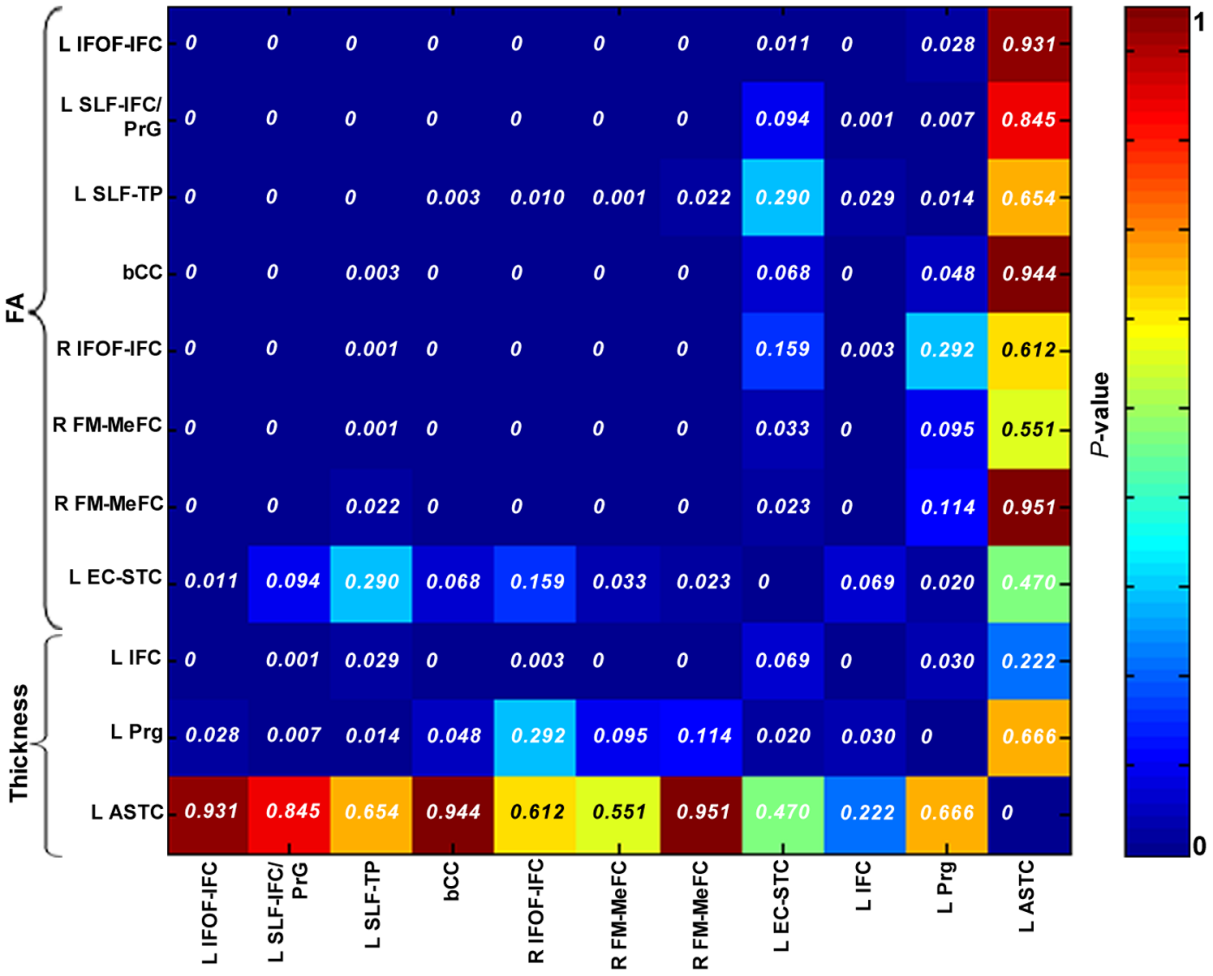
Consistency among WM/GM and between WM and GM matter changes in Group 1. The correlation matrix among all ROIs, including both GM and WM matter, are given.

There was a high level of consistency in the pattern of neural changes among the targeted WM/GM ROIs, but no such consistency between the target ROIs and the control ROIs (*P*<0.05) (Figure 2).

### Consistency between WM and GM changes

As Figure 2 shows, FA of all WM ROIs except for the SLF-TP (*r* = 0.364, *P* = 0.029) showed significant positive correlations with cortical thickness of the IFC (*P*<0.05), but, only FA of the SLF-IFC/Prg and SLF-TP correlated significantly with cortical thickness of the Prg (*r* = 0.442, *P* = 0.007; *r* = 0.405, *P* = 0.014). These results indicated that the change of FA value in the SLF-IFC/Prg was more closely related to the change of cortical thickness than other regions.

An additional analysis showed that GA-FA (averaged from all WM ROIs except for the control ROI of EC-STC) and GA-thickness (averaged from the IFC and Prg) correlated significantly with each other (*r* = 0.586, *P*<0.001), but neither showed significant correlation with the control ROIs (between GA-FA and STC: *r* = 0.008, *P* = 0.964; between GA-thickness and EC-STC: *r* = 0.385, *P* = 0.02 after FDR correction). Consequently, further correlation analyses were not conducted. The regression analysis was conducted next.

**Figure 3:**
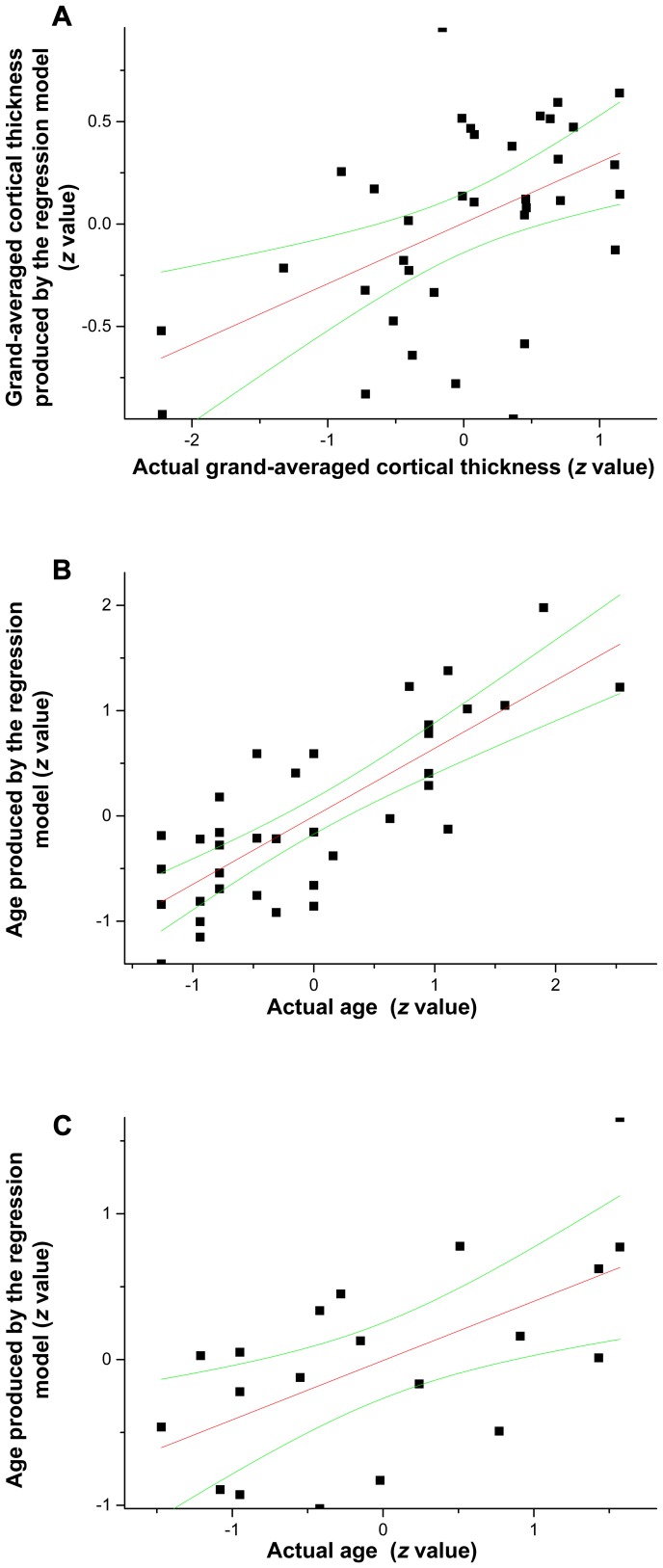
Linear regression fit results. (A) shows fitting result when using grand-averaged cortical thickness as the dependent variable in Group 1. (B) shows fitting result when using age as the dependent variable in Group 1. (C) Re-examination of Group 1's ROI in Group 2's data. Age is the dependent variable. Note that the red line is the linear fit result, whereas the green lines are the confidence internal (95%). For all three panels, the x-axis corresponds to the actual age or cortical thickness, whereas the y-axis corresponds to the age or cortical thickness produced by the regression model.

The regression analysis included L-A-FA (averaged from the SLF-IFC/Prg and IFOF-IFC), L-P-FA (i.e., the SLF-TP), R-FA (averaged from the right IFOF-IFC and FM-MeFC), bCC, and the control ROI of EC-STC as independent variables, and GA-thickness was the dependent variable. The regression model (*F*_(1, 34)_ = 18.301, *P*<0.001) showed that the L-A-FA (*β* = 0.592, *P*<0.001) accounted for 35% of the GA-thickness variance (*R^2^* = 0.35), whereas other WM ROIs did not make significant contributions (Figure 3A). Additional regression analysis using age as the dependent variable showed that the L-A-FA (*β* = −0.815, *P*<0.001) accounted for about 65% of the variance with age (*R^2^* = 0.654, *F*_(1, 34)_ = 67.143, *P*<0.001), whereas other variables did not make significant contributions (Figure 3B).

Together, these results indicated that the dorsal pathway that connects the STC with the IFC showed significant aging-related change in both GM and WM, whereas the ventral pathway and the dorsal pathway connecting the STC with the premotor cortex (i.e., no extension to the IFC) did not. Overall the results supported the LIFO account.

### Re-examination of the neural changes in group two

Results of whole-brain and ROI analyses for group two were provided in the Text S1, Table S1, and Figure S1 and S2. Most importantly, the neural changes (i.e., those in the late maturing dorsal pathway) of group one were examined next using group one's ROIs on FA/cortical thickness for participants in group two. The results showed that the regression analysis that used the L-A-FA, L-P-FA, R-FA and their interactions as the independent variables, and age as the dependent variables, showed significant contributions of the L-A-FA (*β* = −0.669, *P* = 0.001) to the variance with age (*F*_(1, 18)_ = 14.553, *P* = 0.001) which accounted for about 45% of the variance with age (*R^2^* = 0.447) (Figure 3C). No other variables made significant contributions. These results suggest that the L-A-FA made significant contribution to the variance with age, whereas the L-P-FA and the control ROI of EC-STC did not.

## Discussion

This study addressed the theoretical issue about how neural structures that mature at different ages are affected during healthy aging. While LIFO predicts that the neural structures that mature later would be affected more than those that mature earlier, alternative theories such as FIFO predict the opposite pattern. In this study, the results for both groups showed that one of the dorsal language pathways, the one connected by the SLF/arcuate fasciculus between the mid-to-posterior STC and the IFC, was affected more by age with regards to both WM fibers and GM cortical thickness than the other dorsal pathway, the one connecting the STC with the premotor cortex, or the ventral pathway that connects the mid-to-anterior STC with the ventral IFC. These results indicated that the influence of aging on the neural structures serving language is selective, and different neural structures are affected at different stages of aging. Corresponding with this finding, previous evidence has shown that the dorsal and ventral pathways also mature at different ages [15]. While both the ventral pathway connecting the mid-to-anterior STC with the ventral IFC and the dorsal pathway connecting the mid-to-posterior STC and the premotor cortex are clearly present at birth, the other dorsal pathway connecting the STC and the IFC is not [15], [16], [30]. Thus, the present findings suggested that the neural structures which mature later may be affected more than those that mature earlier, supporting the LIFO account of aging for these structures [31].

The above findings were validated in several additional analyses. First, no significant correlations with age were found in the extreme capsule of the ventral pathway. In order to confirm this, two areas in the ventral pathway were selected and WM and GM were obtained (EC-STC for WM and STC for GM). The results did not show any significant correlations between GM/WM and age in these brain areas, either. Second, the brain areas that correlated significantly with age were highly consistent with each other, but not with the control areas which did not correlate significantly with age, in each group's data. These results further indicated that the affected neural structures were driven by similar underlying biological mechanisms and they were distinct from other unaffected neural structures.

The results showed that only FA of the left anterior part of the dorsal pathway (i.e., the part that is specific to the dorsal pathway connecting the STC and the IFC) correlated significantly with cortical thickness in the left IFC and premotor cortex. Meanwhile, FA at this position was also able to account for a large percent of the variance of age as indicated in the regression analysis. Moreover, the close association between the anterior part of the dorsal fiber tract and the cortical thickness/age were replicated in a second independent group of participants. These results help to elucidate the changes in GM and WM during aging, in that they suggest that both WM and GM are affected in ways that are consistent with the LIFO principle for aging.

There is much evidence showing various neural structural change as aging occurs [1], [2], but most of these studies have examined the corpus callosum [4]. One of the robust findings from these studies is that changes in neural structure associated with aging seem to be restricted to the frontal part of the brain, suggesting an anterior-posterior gradient [32]–[34]. Developmental studies have shown that frontal and temporal association areas are the last ones to myelinate [35]. Together the aging and developmental findings are consistent with the assumption that the brain areas that mature last during development tend to decline first during aging. The current results extend this principle to a large-scale neural network corresponding to a high-level cognitive function (i.e., language).

Bartzokis [3] proposed that oligodendrocytes could be one of the mechanisms that underlie the LIFO of aging. Oligodendrocytes produce myelin and changes in myelinization are crucial for human brain development. The axons in the association brain areas myelinate late. It is estimated that the development of these axons reaches its peak at around the fourth decade of life and continue up to the end of the fifth decade of life [10], [36]. The late-differentiating oligodendrocytes cannot produce the same myelin thickness per axon segment as earlier-myelinating oligodendrocytes. The thinner, later-myelinating sheaths are more susceptible to functional impairment and destruction. Thus, later-myelinating neurons of the association areas may be more susceptible to myelin breakdown than earlier-myelinating neurons in the primary motor and visual areas. In addition, this process causes a slow-progressive disruption of neural-impulse transmission that degrades the temporal synchrony of widely distributed neural networks underlying normal brain function. The primary result is large-scale network “disconnection”. The present study provided imaging evidence that is consistent with this proposition.

There are several limitations in this study. First, this study did not include younger age ranges and thus could not examine the trajectory of FA and cortical thickness below fifty years of age [37]. Other studies that have examined younger age groups have shown that the development of WM in the prefrontal, temporal, and parietal areas myelinate late and this continues until the end of the fifth decade of life. Other evidence shows that development of WM peaks at around the fourth decade of life [10], [36]. Moreover, studies have shown decline of both density of GM [38] and FA of WM [10], [36] after age fifty. Thus, it seems that normal aging and late development might partly overlap in some brain areas. Further studies are required to test the development-loss relationship in different brain areas. Second, this study only examined FA of WM and cortical thickness of GM. Future studies are needed to consider whether the development, or aging, process varies with different indexes since different index of neural structures might reflect different aspects of development or aging [39]. Finally, although a cross-sectional design can cover a large age-range, the results need to be addressed using a longitudinal design.

In many countries, the proportion of people aged over 60 years is growing faster than any other age group and this has become a major challenge. There is still much to be learned about the underlying biochemical mechanism of GM/WM changes during aging. In particular, it appears likely that genetic factors may play additional roles in this process. Future studies are needed to quantify the relationship between neural structure changes and the biochemical and genetic factors and how they relate to cognitive functions such as language. Furthermore, intervention approaches may be developed that are designed to delay the aging process.

## Supporting Information

Figure S1 **Neural structural changes with age in Group 2.** (A) shows FA changes. Note that the skeletonized results are “thickened” to help visualization. Left SLF_Prg, left superior longitudinal fasciculus underlying the precentral gyrus; Left SLF_TP, left superior longitudinal fasciculus underlying the temporal-parietal association cortex; Left FM-MeFC, left forceps minor/uncinate fasciculus near the medial frontal cortex (BA10/32); Right SLF-CC, right superior longitudinal fasciculus/anterior corona radiate underlying the cingulate cortex; Right ATR-CC, anterior thalamic radiation close to the cingulate cortex (BA32). (B) shows cortical thickness changes. The colored blobs (blue for cortical thickness, red for FA) indicate brain areas that showed significant negative correlations with age. No positive correlations were found. Left MFC, left middle frontal gyrus (BA10); Left TP, left inferior parietal gyrus (BA39).
(TIF)

Figure S2 **Consistency among WM/GM and between WM and GM matter changes in Group 2.** The correlation matrix among all ROIs, including both GM and WM matter, are given.
(TIF)

Table S1 **Brain regions showing significant correlations with age for Group 2.**
(DOCX)

Text S1 **Imaging data acquisition and results for Group 2.**
(DOC)
