# Supplementary figures and images for: White and Grey Matter Changes in the Language Network during Healthy Aging
Source: PLoS One. 2014 Sep 24;9(9):e108077. doi: 10.1371/journal.pone.0108077 (PMC4176722; doi:10.1371/journal.pone.0108077)

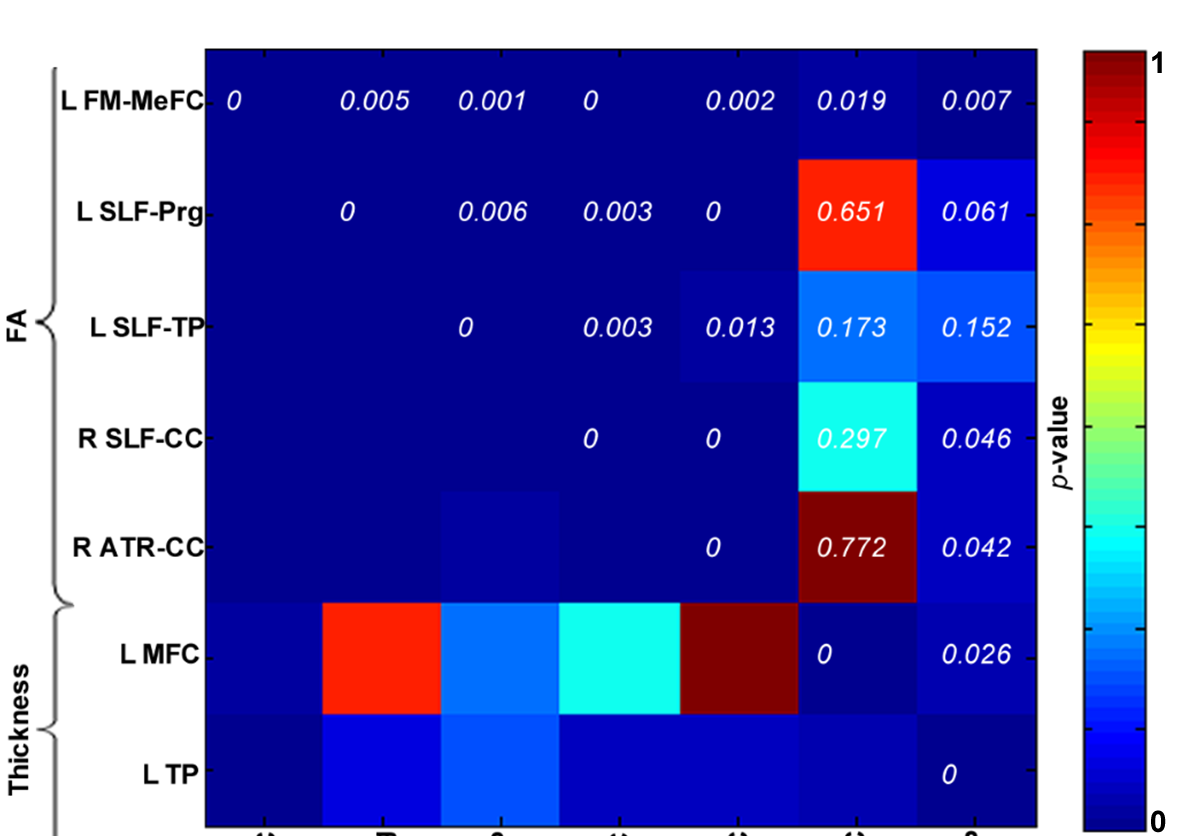

Supplement: Figure S2 — Consistency among WM/GM and between WM and GM matter changes in Group 2. The correlation matrix among all ROIs, including both GM and WM matter, are given. (TIF) [file pone.0108077.s002.tif]
