# Supplementary material for: White and Grey Matter Changes in the Language Network during Healthy Aging
Source: PLoS One. 2014 Sep 24;9(9):e108077. doi: 10.1371/journal.pone.0108077 (PMC4176722; doi:10.1371/journal.pone.0108077)
Supplement: Table S1 — Brain regions showing significant correlations with age for Group 2. (DOCX) [file pone.0108077.s003.docx]

**Table S1 Brain regions showing significant correlations with age.**

| **Brain regions** | **Position** | | | **t-value** | | **Volume**  **(mm^3^/ mm^2^)** | | | |  |
| --- | --- | --- | --- | --- | --- | --- | --- | --- | --- | --- |
|  | **x** | **y** | **z** | |  | | |  | | |
| **Group 2’s white matter (FA) changes** | | | | | | | | | |  |
| Left superior longitudinal fasciculus underlying the precentral gyrus | -44 | -5 | 43 | | 4.775 | | | | 24 | |
| Left superior longitudinal fasciculus underlying the temporal-parietal association cortex | -27 | -44 | 39 | | 5.118 | | | | 26 | |
| Left Forceps minor/Uncinate fasciculus near the medial frontal cortex (BA10/32) | -14 | 34 | -15 | | 5.781 | | | | 29 | |
| Right superior longitudinal fasciculus/  Anterior corona radiate underlying the cingulate cortex | 19 | 27 | 22 | | 5.200 | | | | 156 | |
| Right anterior thalamic radiation close to the cingulate cortex (BA32) | 26 | 36 | 7 | | 4.668 | | | | 29 | |
| **Group 2’s grey matter (cortical thickness) changes** | | | | | | | | | |  |
| Left middle frontal gyrus (BA10) | -22 | 58 | 7 | -3.680 | | | 145 | | |  |
| Left inferior parietal gyrus (BA39) | -45 | -56 | 29 | -2.614 | | | 131 | | |  |
