# Supplementary material for: White and Grey Matter Changes in the Language Network during Healthy Aging
Source: PLoS One. 2014 Sep 24;9(9):e108077. doi: 10.1371/journal.pone.0108077 (PMC4176722; doi:10.1371/journal.pone.0108077)
Supplement: Text S1 — Imaging data acquisition and results for Group 2. (DOC) [file pone.0108077.s004.doc]

**Text S1**

1. **Imaging data acquisition**
   1. ***Group one***

Structural data parameters were: Time repetition (TR) = 1600 ms; time echo (TE) = 2.13 ms; flip angle = 9°; slice thickness = 1 mm; in-plane resolution = 1.0×1.0 mm^2^; 176 interleaved sagittal slices. DTI data: TR = 6 s; TE = 87 ms; slice thickness = 3 mm; in-plane resolution = 2×2 mm; 45 interleaved axial slices; b factor of 1000 s/mm^2^; 12 encoding directions; averaged three times.

- 1. ***Group two***

Structural data parameters were: TR = 1900 ms; TE = 2.13 ms; flip angle = 9°; slice thickness = 1 mm; in-plane resolution = 1.0×1.0 mm^2^; 176 interleaved sagittal slices. DTI data: TR = 11 s; TE = 94 ms; slice thickness = 2 mm; in-plane resolution = 2×2 mm; 65 interleaved axial slices; b factor of 1000 s/mm^2^; 30 encoding directions; averaged three times.

1. **Results for group two**
   1. ***Group two’s DTI results in the whole-brain analysis***

After statistical correction was made, the right SLF/anterior corona radiate underlying the cingulate cortex (SLF-CC) alone showed a significantly negative correlation with age.

To investigate the general correlation pattern further, a statistical threshold of *P* < 0.001 (uncorrected) was used. At this level, the later maturing dorsal fibers connecting the STC with IFC in the left hemisphere correlated negatively with age at two positions: The tract underlying the precentral gyrus (BA6) (SLF-Prg), and the tract underlying the temporal-parietal association cortex (BA39/40) (SLF-TP). Other negative correlations occurred in the forceps minor/uncinate fasciculus near the medial frontal cortex (BA10/32) (FM-MeFC). In the right hemisphere, there were negative correlations with age in the anterior thalamic radiation close to the cingulate cortex (BA32) (ATR-CC). Table S1 and Figure S1A summarize these results.

No significant positive correlations were found in either the left or right hemisphere. Also, no significant correlations were found either in the extreme capsule of the ventral pathway or in the arcuate fasciculus of the other dorsal pathway.

- 1. ***Group two’s cortical thickness results in the whole-brain analysis***

After statistical correction, two left brain areas correlated negatively with age. One area was located in the middle frontal cortex (MFC, BA10), and the other was located in the TP (BA39). These two areas were roughly at the two ends of one of the dorsal pathway. No significant positive correlations were found (Table S1 and Figure S1B).

- 1. ***ROI validation of the aging-related neural changes in group two***

In the regression analysis, all WM ROIs and the interactions between WM ROIs in the left (i.e., SLF-Prg × SLF-TP, SLF-TP × FM-MeFC, SLF-Prg ×FM-MeFC, and SLF-Prg × SLF-TP × FM-MeFC) and right (SLF-CC × ATR-CC) hemispheres were included as independent variable. The analysis produced a model (*F*_(2,19)_ = 23.067, *P* < 0.001) in which the FM-MeFC (*β* = - 0.455, *P* = 0.014) and SLF-CC (*β* = - 0,485, *P* = 0.01) accounted for almost 70% of the variance over age (*R^2^* = 0.699). No other variables made significant contributions. The regression model on the GM ROI (*F*_(1,19)_ = 12.186, *P* = 0.003) showed that the TP (*β* = - 0.635, *P* = 0.003) accounted for 37% of the variance with age (*R^2^* = 0.371), but the MFC and control area (STC) and the interaction between MFC and TP did not make significant contributions. These results suggest that 1) the anterior part of the dorsal pathway (FM-MeFC and SLF-CC ) had a closer relationship with cortical thickness and age than both the posterior part (SLF-TP ) of the dorsal pathway and the ventral pathway (EC-STC), and 2) some of the WM/GM ROIs may correlate with each other, showing high-level consistency in the pattern of neural changes during healthy aging.

- 1. ***Consistency among WM/GM changes in group two***

The FA changes with age were highly consistent across the targeted WM ROIs (i.e., the SLF-Prg, SLF-TP, FM-MeFC, SLF-CC, and ATR-CC) (*P* < 0.05). However, none of these ROIs correlated significantly with the control ROI (EC-STC) (*P* > 0.05). Similar patterns appeared for cortical-thickness measures, that is, there was a significant positive correlation between the MFC and TP (*P* < 0.05), but neither the MFC nor the TP correlated significantly with the control GM ROI (STC) (*P* > 0.05). Thus, it seemed that the affected neural structures had high-level consistency in their patterns of neural changes with age, but did not between them and other neural structures that did not change with age. The correlation matrix is given in Figure S2A.

- 1. ***Consistency between WM and GM changes in group two***

The FA value in the FM-MeFC correlated positively with the cortical thickness of MFC (*r* = 0.52, *P* = 0.019) and TP (*r* = 0.582, *P* = 0.007) (Figure S2A). This suggested that although the FM-MeFC did not lie in the SLF, it might have a close relationship with the late-maturing dorsal pathway. There were no other significant correlations.

Additional analysis showed that the GA-FA and GA-Thickness did not correlate significantly with each other (*P* > 0.05) (Figure S2B). This result indicated that not all WM changes were related to the GM changes of the TP and MFC. The results also showed a significant positive correlation between the L-FA and GA-Thickness (*r* = 0.481, *P* = 0.032), but not between the R-FA and GA-Thickness (*r* = 0.39, *P* = 0.089). Thus, the reliable changes in neural structure appear to be specific to the left hemisphere. Moreover, a significant positive correlation was found between the L-A-FA (obtained by combination of the SLF-Prg and FM-MeFC) and GA-Thickness (*r* = 0.489, *P* = 0.029) but not between the L-P-FA (i.e., the SLF-TP) and GA-Thickness (*r* = 0.337, *P* = 0.146). The FA value in the L-A-FA also correlated positively with the cortical thickness of TP (*r* = 0.567, *P* = 0.009), but the L-P-FA did not (*r* = 0.423, *P* = 0.063). Neither L-A-FA nor L-P-FA correlated significantly with the cortical thickness of MFC (*r* = 0.244, *P* = 0.3; *r* = 0.129, *P* = 0.588). In addition, no significant correlation with GA-Thickness was found with the control area of EC-STC (*r* = 0.138, *P* = 0.56). The EC-STC did not correlate significantly with the MFC (*r* = 0.167, *P* = 0.48) or TP (*r* = 0.082, *P* = 0.73).

The regression analysis confirmed the above pattern. That is, the L-A-FA (*β* = 0.489, *P* = 0.029) accounted for about 20% of the variance of the changes in GA-Thickness (*R^2^* = 0.197, *F*_(1,19)_ = 5.649, *P* = 0.029), whereas other WM ROIs did not make significant contributions. The additional regression analysis showed that the decline in L-A-FA (*β* = -0.792, *P* < 0.001) accounted for about 61% variance with age (*R^2^* = 0.607, *F*_(1,19)_ = 30.305, *P* < 0.001), whereas other variables did not make significant contributions.
